# Supplementary material for: The sprT Gene of Bacillus velezensis FZB42 Is Involved in Biofilm Formation and Bacilysin Production
Source: Int J Mol Sci. 2023 Nov 27;24(23):16815. doi: 10.3390/ijms242316815 (PMC10706128; doi:10.3390/ijms242316815)
Supplement: Supplementary file 1 [file ijms-24-16815-s001.zip › ijms-2725353-supplementary.pdf]

Table S1. Primers used in this study

| oligo | |
|---|---|
| FBO752 | 5’-GCTTGTAAATTCTATCATAATTG-3’ |
| FBO753 | 5’-AGGGAATCATTTGAAGGTTGG-3’ |
| FBO1339 | 5’-ATGGATAACGAACAACTTCAA-3’ |
| FBO1340 | 5’-AAAGTGTCGTTGTCT CTC AAC-3’ |
| FBO1566 | 5’-AAATTCATTGATCGCCACTCC-3’ |
| FBO1567 | 5’-CGACAGTGAACTGCTTGATC-3’ |
| FBO1568 | 5’-CTGATCTGTATCTTTTCGACC-3’ |
| FBO1569 | 5’-GCAATGTCCGCAATTGTTTC-3’ |
| FBO1570 | 5’-TTGATGAGCTGTATGATGTGC-3’ |
| FBO1571 | 5’-CGTCTGCAACCATGACATTC-3’ |
| FBO1572 | 5’-CGAGTATAATGCAAAGGAGG-3’ |
| FBO1573 | 5’-GTCTCTAAAAGAGCGGTCAC-3’ |
| *bacA-F* | 5’-GCGTATGACGGCATTAAGCA-3’ |
| *bacA-R* | 5’-GGGTGGGATACGATGACAGT-3’ |
| *bacB-F* | 5’-GTCCGTCAGAGGAGATACGG-3’ |
| *bacB-R* | 5’-GATGCGGTTCCACGTTTGTA-3’ |
| *bacC-F* | 5’-GTGTATCCACCGTCTGCG-3’ |
| *bacC-R* | 5’-CTCGGCAAGCCTGAAGAA-3’ |
| *bacD-F* | 5’-GTTGGTCGTAATGGCGTCAA-3’ |
| *bacD-R* | 5’-TACTCGGTCGCGGTCATAAA-3’ |
| *bacE-F* | 5’-CCTCACCGTATTCCATTGCG-3’ |
| *bacE-R* | 5’-GACGTTCTGTATCGGCTTCG-3’ |
| *bacF-F* | 5’-GGTTCGGGACTAAGGCGATA-3’ |
| *bacF-R* | 5’-GAAAGAAGCCATCGCGGAAT-3’ |
| *bacG-F* | 5’-CGGCGCCAATATGTTTACCA-3’ |
| *bacG-R* | 5’-AACCCGGGATTCAGACAGTT-3’ |
| *gyrA-F* | 5’-CTAACCCGCCGCCCTTAT-3’ |
| *gyrA-R* | 5’-CTGCGAATGATTTCCAAC-3’ |

Table S2. Protein sequence alignment for *sprT* product and SprT s in some other spieces

| Spieces | Identities % | Positives % | Gaps % |
|---|---|---|---|
| *Bacillus velezensis* DSM 23117 | 100 | 100 | 0 |
| *Bacillus subtilis* 168 | 83 | 89 | 0 |
| *Bacillus licheniformis* | 74 | 84 | 0 |
| *Bacillus pumilus* | 67 | 80 | 2 |
| *Bacillus salacetis* | 65 | 81 | 0 |
| *Bacillus cereus* | 59 | 78 | 0 |
| *Bacillus siamensis* | 97 | 98 | 0 |
| *Bacillus thuringiensis subsp. konkukian* | 59 | 79 | 0 |

| | | | |
|---|---|---|---|
| *Bacillus nakamurai* | 91 | 93 | 0 |
| *Bacillus spizizenii* | 83 | 89 | 0 |
| *Bacillus intestinalis* | 83 | 89 | 0 |
| *Bacillus sp.* EKM208B | 96 | 97 | 0 |
| *Jeotgalibacillus campisalis* | 66 | 81 | 2 |
| *Aeribacillus pallidus* | 63 | 81 | 0 |
| *Priestia koreensis* | 62 | 81 | 0 |
| *Escherichia coli* | 25 | 48 | 2 |
| *Haemophilus influenzae* 86-028NP | 26 | 43 | 6 |
| *Klebsiella pneumoniae* | 25 | 42 | 6 |
| *Pseudomonas ogarae* | 22 | 41 | 8 |
| *Actinobacillus pleuropneumoniae* | 24 | 43 | 10 |
| *Enterobacter hormaechei* | 24 | 44 | 6 |
| *Vibrio parahaemolyticus* | 29 | 43 | 8 |
| *Pectobacterium brasiliense* | 25 | 42 | 10 |
| *Streptococcus equi* | 43 | 66 | 2 |
| *Shewanella xiamenensis* | 24 | 41 | 17 |

Note: Identities stands for protein sequence identity, Positives stands for protein sequence similarity, and Gaps stands for protein sequence gaps that failed to match.
